# Taking the Next Step: Increasing Gait Speed Assessment in Primary Care

**DOI:** 10.1093/geroni/igaf122.119

**Published:** 2025-12-31

**Authors:** Lisa Barry, Kathryn Callahan, Mariana Wingood, Richard Fortinsky, Karina Berg, Natong Lin, Song Han, Nicholas Pajewski

**Affiliations:** University of Connecticut, Farmington, Connecticut, United States; Wake Forest University School of Medicine, Winston-Salem, North Carolina, United States; Wake Forest University School of Medicine, Winston-Salem, North Carolina, United States; University of Connecticut, Farmington, Connecticut, United States; UConn Health, Farmington, Connecticut, United States; University of Connecticut, Farmington, Connecticut, United States; University of Connecticut, Farmington, Connecticut, United States; Wake Forest University School of Medicine, Winston-Salem, North Carolina, United States

## Abstract

Gait speed is a simple and robust predictor of outcomes in older persons including disability and falls. Yet, despite widespread acceptance as an important clinical indicator, it is not routinely assessed during office visits. We sought to implement gait speed assessment into Medicare Annual Wellness Visit (AWV) workflow at four clinical practices across two healthcare systems. Gait speed was assessed using a previously-validated, radio frequency identification (RFID) technology-based system. Patients complete a 4-meter walk while wearing an armband containing an RFID tag. Wall-mounted antennae identify the tag and a computer program calculates gait speed (meters per second). We assessed implementation feasibility and fidelity using a mixed methods approach informed by the Consolidated Framework for Implementation Research 2.0. Implementation strategies included: identifying clinical champions; developing a gait speed field in Epic, the electronic medical record system; training medical assistants to conduct gait speed assessments before “rooming” patients; and assessing the proportion of AWVs where the system worked as intended. Preliminary results are available from the first clinic. Over the first 6 weeks of the 12-week implementation period, we observed 35 AWVs. In the 3-week first phase, medical assistants successfully assessed gait speed and recorded results into Epic during 16/23 (70%) of AWVs. After medical assistant re-training and re-programming range adjustment to optimize tag detection, gait speed was successfully assessed in 100% of the 12 visits observed in the 3-week second phase. Our findings provide encouraging evidence indicating that gait speed assessment using RFID technology can be implemented into AWV workflow.

